# Improved Accuracy and Safety of Pedicle Screw Placement by Using a Probe with an Electrical Conductivity-Measuring Device during Severe Syndromic and Neuromuscular Scoliosis Spine Surgery

**DOI:** 10.3390/jcm11020419

**Published:** 2022-01-14

**Authors:** Takashi Yurube, Yutaro Kanda, Masaaki Ito, Yoshiki Takeoka, Teppei Suzuki, Koki Uno, Ryosuke Kuroda, Kenichiro Kakutani

**Affiliations:** 1 Department of Orthopaedic Surgery, Kobe University Graduate School of Medicine, 7-5-1 Kusunoki-cho, Chuo-ku, Kobe 650-0017, Japan; youthfuldays_y_k@yahoo.co.jp (Y.K.); yoshiki_tkk@hotmail.com (Y.T.); kurodar@med.kobe-u.ac.jp (R.K.); kakutani@med.kobe-u.ac.jp (K.K.); 2 Department of Orthopaedic Surgery, National Hospital Organization Kobe Medical Center, 3-1-1 Nishiochiai, Suma-ku, Kobe 654-0155, Japan; maito28710@yahoo.co.jp (M.I.); teppeisuzuki@hotmail.com (T.S.); uno.koki.hy@mail.hosp.go.jp (K.U.)

**Keywords:** syndromic scoliosis, neuromuscular scoliosis, electrical conductivity-measuring probing device, pedicle screw placement, segmental fixation, deformity, spine

## Abstract

An electrical conductivity-measuring device (ECD) has recently been developed to support pedicle screw placement. However, no evidence exists regarding its efficacy for syndromic/neuromuscular scoliosis with extremely difficult screwing. We retrospectively reviewed 2010–2016 medical records of 21 consecutive syndromic/neuromuscular scoliosis patients undergoing free-hand segmental fixation surgery at our institution and compared the pedicle screw insertion accuracy and safety between 10 with a conventional non-ECD probe (2010–2013) and 11 with an ECD probe (2014–2016). We analyzed preoperative pedicle shape and postoperative screw placement in computed tomography. There were no significant differences between ECD and non-ECD groups in demographic, clinical, and treatment characteristics including scoliosis severity and pedicle diameter. The abandonment rate due to liquorrhea or perforation was lower in ECD (12.3%) than in non-ECD (26.7%) (*p* < 0.01). Acceptable insertion without perforation or <2-mm lateral/cranial position was more frequent in ECD (67.1%) than in non-ECD (56.9%) (*p* = 0.02). Critical ≥5-mm medial/caudal malposition was not seen in ECD (0.0%) but in non-ECD (2.4%) (*p* = 0.02). The perforation distance was shorter in ECD (2.2 ± 1.1 mm) than in non-ECD (2.6 ± 1.7 mm) (*p* = 0.01). Results involve small sample size, selection, performance, and learning curve biases; nevertheless, ECD could be useful for more accurate and safer pedicle screw placement in severe syndromic/neuromuscular scoliosis.

## 1. Introduction

Instrumentation of the posterior spine with pedicle screw is currently the most common surgical technique to stabilize, fix, and correct pathological changes of the spine caused by degenerative disease, developmental scoliosis, trauma, and tumors [1,2,3]. However, the rate of pedicle screw misplacement has been reported from 5% to 41% in the lumbar spine and from 3% to 55% in the thoracic spine [1]. Moreover, scoliosis patients have complicated morphometric characteristics such as asymmetrical vertebrae and vertebral rotations, which increased the risk of pedicle screw malposition [4]. The classification based on the etiology, provided by the Scoliosis Research Society (https://www.srs.org/, accessed on 16 April 2019), is often used to diagnose and treat scoliosis. While idiopathic scoliosis is the most common, definite causes including congenital scoliosis and syndromic/neuromuscular scoliosis account for 20% to 25% [5]. Particularly in patients with syndromic scoliosis and those with neuromuscular scoliosis, pedicle screw placement can further be challenging even for experienced surgeons because of severe spinal deformity, poor bone quality, and narrowed or diminished pedicles [3,6,7]. Pedicle screw misplacement with the perforation of the pedicle cortex facilitates the loss of stability and correction, which can ultimately lead to the most serious spine-related condition of spinal cord and/or nerve root injuries when extensive mediocaudal breakage of the pedicle wall occurs [8,9,10].

Various methods have currently been utilized to insert screws into the pedicle accurately. Traditional free-hand pedicle screw placement based on the anatomical landmarks is often used [2,3]. However, this method is highly dependent on the surgeon’s experience and expertise [11]. Intraoperative fluoroscopic imaging is widely used to visualize the pedicle bones and also to evaluate the position of inserted pedicle screws [12]. However, unsatisfactory rates of misplacement and related complications have been reported in several systematic reviews [1,9]. In addition, there is a serious concern about excessive radiation exposure to both surgical staffs and patients [13]. Intraoperative navigation systems are helpful to assist with accurate pedicle screw placement. Many studies using image-navigated techniques have showed a favorable efficacy to avoid pedicle screw misplacement [1,14,15]. However, extended operation time with multiple scans is necessary during long segmental fusion surgery [16]. The implementation of the systems indeed requires enormous equipment costs. Moreover, these techniques still have limitations including the learning curve and calibration errors [17].

A probe with an electrical impedance conductivity-measuring device (ECD) has recently been developed to improve the accuracy of pedicle screw placement [18,19]. The ECD probe can be used as a mechanical tool as well as a conventional probe and enables surgeons to detect pedicle breaches in real time and to redirect the probe away from potential cortical breaches by monitoring the electrical conductivity in the surrounding tissues, which alters depending on the tissue type. This device uses audio alerts and light-emitting diode warning signals to guide surgeons. According to a limited number of papers [16,20,21,22,23,24,25,26,27,28], the usefulness of ECD for pedicle proving has been reported during posterior fusion surgery for scoliosis, degenerative disease, and tumors. However, no evidence exists regarding its efficacy even for syndromic and neuromuscular scoliosis in which pedicle screw placement is extremely difficult. Thus, to clarify the contribution of the ECD probe to the accuracy and safety of pedicle screw placement in patients with severe syndromic/neuromuscular scoliosis, a retrospective comparative study between the initial consecutive 10 cases with a conventional probe and the last consecutive 11 cases with the ECD probe was designed.

## 2. Materials and Methods

### 2.1. Study Design and Ethics Statement

This was a retrospective case–control study. Data were collected from historical medical records under the approval and guidance of the Institutional Review Board (IRB) at Kobe University Graduate School of Medicine (No. B190002, 16 April 2019 approval). Considering the nature of the retrospective study design to review medical records of patients who completed the treatment, IRB waived the requirement to obtain informed consent. However, for the use of personal information and images of patients, written informed consent for publication was obtained from each patient in accordance with the principles of the Declaration of Helsinki and the laws and regulations of Japan.

### 2.2. Patients

Twenty-three consecutive patients with syndromic/neuromuscular scoliosis who underwent posterior segmental fixation surgery from 2010 to 2016 at our university hospital were included in this study. Anterior surgery and revision surgery were excluded. Surgical indications were generally progressive scoliosis with the Cobb angle of >60° and/or difficulty to stay in a sitting position requiring pelvic fixation. Clinically, pulmonary function was also taken into account. In fact, we surgically treated all consecutive cases of referred patients with syndromic/neuromuscular scoliosis fulfilling surgical indications and did not refuse surgery because of the worsened general condition or high risk of anesthesia. In Japan, preoperative and postoperative scans of computed tomography (CT) were still acceptable when needed with minimized radiation exposure, which was therefore performed in all scoliosis cases without any selection bias. Demographics of patients regarding age, sex, underlying diseases, clinical characteristics regarding the preoperative Cobb angle and pedicle diameter in images of standing or sitting (difficult to stand up in many cases) thoracic and lumber spine radiography and CT, and treatment characteristics regarding the postoperative Cobb angle, correction rate, number of fixed vertebrae, operation time, and blood loss were investigated. Two patients were excluded because of one case with anterior fusion surgery and one case with revision surgery. Consequently, 21 patients (age, 21.5 ± 11.0 years; 11 men and 10 women) were finally included. Underlying diseases of these patients are listed in Table 1, the most common of which was cerebral palsy (*n* = 5).

### 2.3. Surgical Procedures, Exposures, and Comparisons

Free-hand posterior segmental pedicle screw fixation was performed by the single board-certified senior surgeon using the single instrumentation system with the minimum 4.5-mm screw diameter (Mesa^®^ Deformity Spinal System; K2M/Stryker, Leesburg, VA, USA). Ponte osteotomy was performed at six periapical intervertebral segments. If necessary, fixation down to the pelvis using iliac or S2 alar–iliac screws was applied. Intraoperative monitoring of spinal cord function was conducted by motor evoked potentials. Even in pedicles with the outer diameter of <4.5 mm, we attempted screw placement based on the pedicle expansion concept and in-out-in screw insertion technique [10]. After the pilot hole preparation and intrapedicular marker insertion, intraoperative fluoroscopic imaging was applied; however, clear visualization of pedicle tunnels was difficult because of advanced deformity, curve, and rotation. We finally placed screws only when the maintenance of intra-vertebral bone position was confirmed without recognizable liquorrhea or medial pedicle wall perforation. If difficult, alternative fixation by the single or combined use of laminar hooks, transverse process hooks, and sublaminar tapes was applied. A screw with the largest diameter that could be inserted into the pedicle or alternatively vertebra was selected from preoperative CT examination. In cases with the outer pedicle diameter of <4.5 mm, 4.5-mm diameter screw was inserted. Then, as the exposure to a change in the treatment, a pedicle probe with an ECD (PediGuard^®^; SpineGuard, Vincennes, France) was used. The use of ECD was approved from 13 August 2013 in Japan and from 1 November 2013 at our institution; therefore, we tested and decided to apply it for all cases of syndromic and neuromuscular scoliosis surgery from 1 January 2014. Further, we compared findings between patients whose pedicle screws were inserted with a conventional probe by a free-hand technique as the non-ECD group (2010–2013) and those whose screws were placed with PediGuard^®^ as the ECD group (2014–2016). Consequently, the non-ECD group comprised 288 pedicles from the initial consecutive cases of 10 patients (age, 20.5 ± 7.0 years; 5 men and 5 women) while the ECD group consisted of 284 pedicles from the last consecutive cases of 11 patients (age, 22.4 ± 14.0 years; 6 men and 5 women).

### 2.4. Radiological Evaluations and Outcome Measures

Preoperative pedicle morphology and postoperative screw placement were evaluated on CT multiplanar reconstruction images. First, at pre-operation, the shape and outer diameter of all target pedicles were measured. Then, at post-operation, the achievement and abandonment rates of pedicle screw insertion were investigated, calculated as the number of screw-placed/unplaced pedicles/the number of instrumentation-planned pedicles (2 × instrumentation-planned vertebrae). Further, the position of inserted pedicle screws was assessed through axial, sagittal, and coronal plane images to determine whether pedicle wall breaches occurred medially, laterally, caudally, or cranially and then considered correct if screws were completely placed within the pedicle (intrapedicular position). If not within the pedicle, screw placement was categorized based on the deviation direction and distance. Based on the ECD primary nature of avoiding neural injury, laterally or cranially deviated screws of <2 mm were considered as in a relatively acceptable position whereas laterally or cranially deviated screws of ≥2 mm were determined as in a mechanically threatening malposition based on the low fixability. Meanwhile, any medially or caudally deviated screws were judged as in a neurologically threatening malposition. Regarding the degree of deviation, pedicle breaches were classified by grades based on 2-mm or 3-mm increments and pedicle screws with breaches <2 mm were recognized as in a safe zone in prior studies [8,29]. Therefore, the cut-off value of pedicle breaches was set at 2 mm and 5 mm in this study. The specific perforation distance was also examined. Measurements of pedicle diameter and screw misplacement length were performed twice at a one-week interval by each of three spine surgeon examiners who were blinded to this study, and the mean values were used for evaluation.

### 2.5. Statistical Analysis

All statistical analyses were performed using IBM SPSS Statistics 23.0 (IBM, Armonk, NY, USA) with significance set at a *p*-value of <0.05. The Student’s *t*-test after normality assumption and the Pearson’s chi-squared test or Fisher’s exact test were used for the comparison between ECD and non-ECD groups of continuous variables and of categorical variables, respectively. In addition, the intra-class correlation coefficient was calculated to clarify the intra-observer and inter-observer reliability for CT measurements.

## 3. Results

### 3.1. Demographic, Clinical, Treatment, and Pedicle Morphological Characteristics of Patients with Syndromic/Neuromuscular Scoliosis

Between the two syndromic/neuromuscular scoliosis patient groups of ECD (*n* = 11) and non-ECD (*n* = 10), there were no significant differences in the patient age, sex, preoperative Cobb angle, postoperative Cobb angle, correction rate, and blood loss (Table 2). Although not statistically significant, trends toward the decrease in the number of fixed vertebrae and in the operation time were observed in the ECD group compared to in the non-ECD group (Table 2). No significant differences were also observed in the pedicle diameter of the thoracic spine as well as of the lumbar spine (Table 3). In CT examination, the intra-observer reliability by the intra-class correlation coefficient for preoperative pedicle diameter and postoperative screw misplacement length was 0.86–0.90 and 0.84–0.89, respectively. Similarly, the inter-observer reliability was 0.93 and 0.91. The repeatability and reproducibility of both measurements were acceptable.

### 3.2. Pedicle Screw Placement Frequency, Accuracy, and Safety of Patients with Syndromic/Neuromuscular Scoliosis

**Figure 1:**
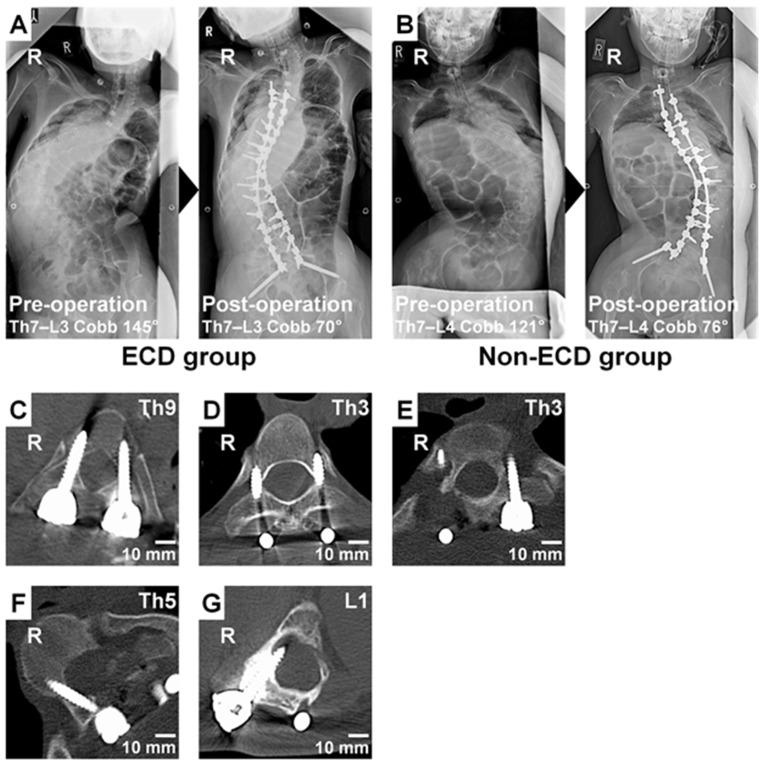
(**A**,**B**) Anteroposterior whole spine radiographs at pre-operation and post-operation of a 23-year-old man with neuromuscular paralysis and scoliosis by astrocytoma in the electrical conductivity-measuring device (ECD) group (**A**) and 24-year-old woman with neuromuscular paralysis and scoliosis by cerebral palsy in the non-ECD group (**B**). The abandonment rate of pedicle screw placement was slightly lower by using a probe with ECD than by using a conventional probe. (**C**) An axial computed tomographic (CT) image of a 24-year-old woman with neuromuscular paralysis and scoliosis by cerebral palsy. Successful screw placement with the intrapedicular position in every direction was observed at Th9. (**D**) An axial CT image of an 18-year-old man with von Recklinghausen’s neurofibromatosis type I and scoliosis. The enlarged spinal canal, narrowed pedicles, and relatively acceptable pedicle screw placement located <2 mm laterally were observed with mild dural ectasia at Th3. (**E**) An axial CT image of a 16-year-old man with the Marfan syndrome and scoliosis. Mechanically critical pedicle screw misplacement located >5 mm laterally was observed at Th3. (**F**) An axial CT image of a 16-year-old man with the Marfan syndrome and scoliosis. Neurologically threatening pedicle screw misplacement located ≥2 mm medially was observed at Th5. (**G**) An axial CT image of a 14-year-old woman with neuromuscular paralysis and scoliosis by cerebral palsy. Neurologically critical pedicle screw misplacement located ≥5 mm medially was observed at L1. In all images, R indicates the right side of the body.

The preparation of pilot screw holes was performed at 288 pedicles of the non-ECD group with a free-hand technique and at 284 pedicles of the ECD group with the aid of electrical conductivity-measuring PediGuard^®^. The ECD probe reduced the abandonment rate of pedicle screw insertion due to abnormal bleeding, recognizable liquourrhea, or identifiable perforation (12.3% in ECD versus 26.7% in non-ECD, *p* < 0.01) (Table 4, Figure 1A,B). Postoperative CT analysis then identified a relatively maintained accuracy of pedicle screw placement with and without ECD, showing no significant difference in the non-perforation rate of pedicles in every direction (50.2% in ECD versus 42.2% in non-ECD, *p* = 0.09) (Table 5, Figure 1C). Regarding lateral and cranial perforations, acceptable pedicle screw placement without perforation or located <2 mm laterally or cranially was more frequently observed in the ECD group (67.1%) than in the non-ECD group (56.9%) (*p* = 0.02) (Table 5, Figure 1D). There was no significant difference in pedicle screw placement located laterally or cranially between the two groups (31.7% in ECD versus 37.4% in non-ECD, *p* = 0.20). However, mechanically threatening pedicle screw misplacement located ≥2 mm laterally or cranially was observed in 37 pedicles (14.9%) of the ECD group but in 48 pedicles (22.7%) of the non-ECD group (*p* = 0.03) (Table 5). Mechanically critical lateral or cranial perforation of ≥5 mm was also seen in 4 pedicles (1.6%) of the ECD group but in 11 pedicles (5.2%) of the non-ECD group (*p* = 0.04) (Table 5, Figure 1E). Regarding medial and caudal perforations, there was no significant difference between the two groups (18.1% in ECD versus 20.4% in non-ECD, *p* = 0.53) (Table 5). Neurologically threatening pedicle screw misplacement located ≥2 mm medially or caudally was also observed in 8 pedicles (3.2%) of the ECD group and in 11 pedicles (5.2%) of the non-ECD group (*p* = 0.28) (Table 5, Figure 1F). However, neurologically critical medial or caudal perforation of ≥5 mm was seen in no pedicles (0.0%) of the ECD group but in 5 pedicles (2.4%) of the non-ECD group (*p* = 0.01) (Table 5, Figure 1G). In addition, the perforation distance was significantly shorter in the ECD group (2.2 ± 1.1 mm) than in the non-ECD group (2.6 ± 1.7 mm) (*p* = 0.02). In fact, there were no patient cases requiring revision surgery due to pedicle screw misplacement.

### 3.3. Technical Note

**Figure 2:**
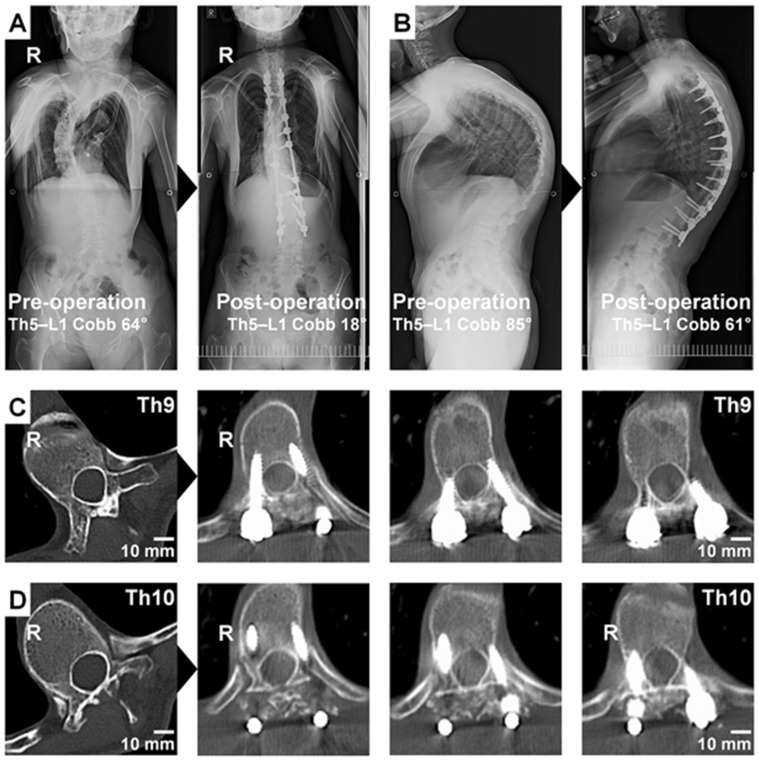
A technical note of a probe with an electrical conductivity-measuring device (ECD) in a 20-year-old man with the Sotos syndrome and scoliosis. (**A**,**B**) Anteroposterior (**A**) and lateral (**B**) whole spine radiographs at pre-operation and post-operation. His right-curved scoliosis and kyphosis at Th5–L1 were surgically treated. (**C**,**D**) Axial CT images at pre-operation and post-operation. The diameter of left concave side’s pedicles at Th9 (**C**) and Th10 (**D**), the apical vertebrae of scoliosis, was severely reduced because of the deformity. The ECD probe was useful to place pedicle screws safely without perforation of the medial wall of left Th9 and Th10 pedicles. Consequently, in-out-in pedicle screw insertion was accomplished at the left side of both segments under the ECD guidance of audio alerts and light-emitting diode warning signals. In all images, R indicates the right side of the body.

A 20-year-old man with the Sotos syndrome presented at pre-operation right-curved scoliosis with the Cobb angle of 64° and severe kyphosis with the Cobb angle of 85° at Th5–L1 (Figure 2A,B). We performed posterior segmental fixation surgery at Th4–L3 with the aid of the ECD probe with a curved tip. In this patient, left concave side’s pedicles at Th9 (Figure 2C) and Th10 (Figure 2D), the apical vertebrae of scoliosis, was remarkably narrowed because of the severe deformity. The ECD probe’s guidance of audio alerts and light-emitting diode warning signals was useful for safe pedicle screw placement without any violation of the spinal canal. First, the ECD signal was medium in the pitch and cadence when the curved tip toward the lateral position was within the posterior pedicle’s cancellous bone. Then, the signal became higher (but not as high as the medial wall perforation) when the curved tip toward the lateral position was possibly located outside the pedicle at the intermediate depth calculated by preoperative CT. Finally, when the curved tip turned toward the medial position and went forward to the intra-vertebral body’s cancellous bone, the signal became low to medium again. Specifically, the ECD signal could help surgeons estimate the depth, direction, and position of the probe tip, e.g., by rotating, which does not require any extra effort to alter their technique for pedicle screw placement. At post-operation, his right-curved scoliosis and kyphosis at Th5–L1 were treated up to 18° and 61°, respectively (Figure 2A,B). Consequently, in-out-in insertion of pedicle screws with the retention of the medial wall of left Th9 and Th10 pedicles was accomplished (Figure 2C,D).

## 4. Discussion

In scoliosis surgery, the use of all-pedicle screw constructs has become common. The frequency and accuracy of pedicle screw placement are essential for acquiring satisfactory surgical outcomes. Most scoliosis studies regarding the accuracy and safety of pedicle screw insertion represent idiopathic scoliosis. The perforation rate of free-hand pedicle screw placement ranged from 3.7% to 65% [2,30]. A retrospective study including 2020 pedicle screws from 140 patients with adolescent idiopathic scoliosis reported that the overall perforation rate of free-hand pedicle screw placement was 20.3% [30]. In neuromuscular scoliosis, a retrospective study of 1009 pedicle screw placement by a free-hand technique reported mispositioned pedicle screws in 27% [31]. In patients with the Marfan syndrome, 241 of 783 (30.8%) pedicles were breached by a free-hand technique [6]. These findings suggest that pedicle screw placement in syndromic and neuromuscular scoliosis is highly challenging even for experienced surgeons due to dystrophic pedicles and dural ectasia. Therefore, it is desirable to use any guidance tools especially in patients with syndromic/neuromuscular scoliosis.

Prior reports studying the ECD probe in scoliosis have shown its efficacy during surgery. The ECD increased pedicle screw placement accuracy and decreased insertion time and radiation exposure in posterior adolescent idiopathic scoliosis surgery [21]. A study using the ECD during surgery for adolescent idiopathic, congenital, syndromic, and neuromuscular scoliosis demonstrated a reduction in the incidence of intraoperative neuromonitoring alarm and improvement in the safety of pedicle screw placement, although more than half of the subjects consisted of idiopathic scoliosis patients [24]. Another study using a fluoroscopy, three-dimensional printed model, ECD, and intraoperative CT navigation system in 31 patients with idiopathic (*n* = 28), syndromic (*n* = 2), and congenital (*n* = 1) scoliosis also demonstrated an acceptable accuracy and prevention from critical misplacement of the ECD, which was comparable with the three-dimensional model and intraoperative navigation system [16]. However, ECD efficacy for syndromic and neuromuscular scoliosis surgery requiring much more difficult screwing due to severe deformity and pedicle narrowing has not been fully investigated. The current study presents the primary usefulness of ECD for more accurate pedicle probing and higher density placement with a reduced incidence and severity of screw malposition during even complicated syndromic and neuromuscular scoliosis surgery. Then, as shown in the Technical Note (Figure 2C,D), ECD with a curved tip could be useful to facilitate safer screw insertion by avoiding critical medial pedicle wall perforation through the intraoperative dynamic detection. However, currently the ECD cannot clearly distinguish the type of existing tissues at the deeper area of bone breaches: muscles, blood vessels, nerve roots, and the dural tube. Further development and investigation are required.

In our study series, pedicle screws were inserted using either with a free-hand technique or with the ECD assistance, which can help surgeons to detect pedicle breaches. In both groups, subjects had severe deformity with the mean thoracic pedicle diameter of <3.5 mm and the mean preoperative Cobb angle of >80°. Under these severe circumstances, more than half of pedicle screw placement by a free-hand technique led to the pedicle perforation in every direction. Inserted pedicle screws with breaches <2 mm were considered as a safe zone in many studies [8,29]. However, even if medial and caudal breaches are <2 mm, the dural tube and spinal cord can be close to the concave side of pedicles in patients with severe syndromic/neuromuscular scoliosis. Regarding lateral and cranial perforations, although relatively safe, breaches may lead to the loss of the stability. Therefore, we determined intrapedicular placement and lateral or cranial perforation of <2 mm as an acceptable position and lateral or cranial perforation of ≥2 mm as a mechanically threatening malposition. Meanwhile, any medial or caudal perforation was considered as a neurologically threatening malposition. In particular, medial or caudal perforation of ≥5 mm was judged as critical. Our results suggest that the ECD probe could be helpful to improve the frequency and accuracy of pedicle screw placement and also to avoid mechanical property deterioration and critical misplacement in every direction. Although we cannot ignore the variation in patient and disease characteristics and the presence of the learning curve, these findings could lead to the decreased operation time in patients with ECD [21,24].

This study has several limitations. First, this study cases and controls were retrospectively collected from not randomly distributed, consecutive case series; therefore, potential selection bias exists despite no significant differences in patient demographics and clinical and treatment characteristics related to screw placement between ECD and non-ECD groups. In addition, the learning curve would affect results because we selected patients who underwent surgery before the appearance of the ECD probe as the historical control; consequently, performance bias could overestimate the efficacy of ECD. Second, the enrolled subjects were relatively small in number and heterogeneous in patient demographics and clinical characteristics because of the rarity of diseases and nature of the retrospective design. Third, the comparison in the ECD efficacy with other surgery-supporting methods capable of facilitating accurate pedicle screw placement, e.g., intraoperative navigation systems and three-dimensional printed models [16], was not performed in this study due to limited equipment availability. However, the ECD probe should have advantages in terms of easy handling, direct repositioning, and reduced radiation exposure without extra expense and time-consuming setup.

## 5. Conclusions

This retrospective case–control study provides only limited evidence because of a high-risk involvement of bias with the small sample size and learning curve effects; nevertheless, the ECD probe could be a helpful support tool to provide more accurate and safer pedicle screw placement, leading to substantial reductions in mechanical instability, critical malposition, and then potential neural damage, even during challenging syndromic/neuromuscular scoliosis spine surgery. Future prospective clinical trials are warranted to clarify the efficacy of ECD.

## Figures and Tables

**Table 1:** Underlying diseases of 21 patients with syndromic/neuromuscular scoliosis.

	*n* (%)
Cerebral palsy	5 (23.8)
Marfan syndrome	4 (19.0)
Neurofibromatosis type I/von Recklinghausen	3 (14.3)
Muscle dystrophy	1 (4.8)
Loeys–Dietz syndrome	1 (4.8)
Sotos syndrome	1 (4.8)
Congenital myopathy	1 (4.8)
Escobar syndrome	1 (4.8)
Arthrogryposis multiplex congenita	1 (4.8)
Astrocytoma	1 (4.8)
Osteoblastoma	1 (4.8)
Prune belly syndrome	1 (4.8)

**Table 2:** Demographic, clinical, and treatment characteristics of 21 patients undergoing segmental fixation surgery with and without an electrical conductivity-measuring probing device.

	Patients Undergoing Surgery with ECD	Patients Undergoing Surgery with Non-ECD	*p*
	(*n* = 11)	(*n* = 10)	
Age, mean ± SD [years]	22.4 ± 14.0	20.5 ± 7.0	0.71 *
Male/female sex, *n* (%)	6 (54.5)/5 (45.5)	5 (50.0)/5 (50.0)	0.84 **
Preoperative Cobb angle, mean ± SD [°]	83.4 ± 26.0	82.3 ± 28.0	0.92 *
Postoperative Cobb angle, mean ± SD [°]	41.8 ± 18.4	45.7 ± 23.4	0.67 *
Correction rate of the Cobb angle, mean ± SD [%]	49.7 ± 18.3	46.5 ± 18.6	0.70 *
Number of the fixed vertebrae, mean ± SD [no.]	11.9 ± 1.5	13.4 ± 1.8	0.05 *
Operation time, mean ± SD [min]	478.5 ± 97.5	596.8 ± 205.0	0.10 *
Blood loss, mean ± SD [g]	2489.7 ± 2020.3	2872.2 ± 1294.6	0.62 *

*, ** Calculated by the Student’s *t*-test (*) or Pearson’s chi-squared test (**). ECD, electrical conductivity-measuring device; SD, standard deviation.

**Table 3:** Morphological characteristics of 572 pedicles undergoing the preparation of pilot holes for screw with and without an electrical conductivity-measuring probing device.

	Pedicles Undergoing Screw Hole Preparation with ECD	Pedicles Undergoing Screw Hole Preparation with Non-ECD	*p*
	(*n* = 284)	(*n* = 288)	
Diameter in the thoracic spine, mean ± SD [mm]	3.14 ± 1.82	3.44 ± 1.98	0.13 *
Diameter in the lumbar spine, mean ± SD [mm]	5.52 ± 2.90	4.93 ± 2.77	0.16 *

* Calculated by the Student’s *t*-test (*). ECD, electrical conductivity-measuring device; SD, standard deviation.

**Table 4:** Screw placement frequency of 572 pedicles undergoing the preparation of pilot holes with and without an electrical conductivity-measuring probing device.

	Pedicles Undergoing Screw Hole Preparation with ECD	Pedicles Undergoing Screw Hole Preparation with Non-ECD	*p*
	(*n* = 284)	(*n* = 288)	
Screw placement, *n* (%)	249 (87.7)	211 (73.3)	<0.01 *^,†^

* Calculated by the Pearson’s chi-squared test (*). ^†^ *p* < 0.05. ECD, electrical conductivity-measuring device.

**Table 5:** Screw placement accuracy and safety of 460 pedicles undergoing the insertion with and without an electrical conductivity-measuring probing device.

	Pedicles Undergoing Screw Placement with ECD	Pedicles Undergoing Screw Placement with Non-ECD	*p*
	(*n* = 249)	(*n* = 211)	
Acceptable pedicle screw placement			
Intrapedicular position, *n* (%)	125 (50.2)	89 (42.2)	0.09 *
Intrapedicular or <2-mm lateral/cranial position, *n* (%)	167 (67.1)	120 (56.9)	0.02 *^,†^
Mechanically threatening pedicle screw misplacement			
≥2-mm lateral/cranial malposition, *n* (%)	37 (14.9)	48 (22.7)	0.03 *^,†^
≥5-mm lateral/cranial malposition, *n* (%)	4 (1.6)	11 (5.2)	0.04 **^,†^
Neurologically threatening pedicle screw misplacement			
Medial/caudal malposition, *n* (%)	45 (18.1)	43 (20.4)	0.53 *
≥2-mm medial/caudal malposition, *n* (%)	8 (3.2)	11 (5.2)	0.28 *
≥5-mm medial/caudal malposition, *n* (%)	0 (0.0)	5 (2.4)	0.02 **^,†^

*, ** Calculated by the Pearson’s chi-squared test (*) or Fisher’s exact test (**). ^†^ *p* < 0.05. ECD, electrical conductivity-measuring device.

## Data Availability

The data presented in this study are available on reasonable request from the corresponding author.
